# RETRACTED: Davarpanah, A. Parametric Study of Polymer-Nanoparticles-Assisted Injectivity Performance for Axisymmetric Two-Phase Flow in EOR Processes. *Nanomaterials* 2020, *10*, 1818

**DOI:** 10.3390/nano16040235

**Published:** 2026-02-12

**Authors:** Afshin Davarpanah

**Affiliations:** Department of Mathematics, Aberystwyth University, Aberystwyth SY23 3FL, UK; afd6@aber.ac.uk

The journal retracts the article “Parametric Study of Polymer-Nanoparticles-Assisted Injectivity Performance for Axisymmetric Two-Phase Flow in EOR Processes” [1] cited above.

Following publication, concerns were raised with the Editorial Office regarding the integrity of several figures in this publication [1].

Adhering to our standard Journal procedure, an investigation was conducted by the Editorial Office and Editorial Board that confirmed the similarity of graph lines between Figures 7 and 8, and duplication between Figures 9a and 10a. While the author collaborated during the process, he could not provide a feasible explanation about the duplications and the supporting materials submitted were incomplete and could not be used to verify the validity of the data and findings in the publication [1]. Additionally, the investigation found a high number of self-citations, which do not comply with MDPI’s citation policy (https://www.mdpi.com/ethics#_bookmark20).

Consequently, the Editorial Board has lost confidence in the reliability of the findings, and has decided to retract this publication, as per MDPI’s retraction policy (https://www.mdpi.com/ethics#_bookmark30).

This retraction was approved by the Editor-in-Chief of the journal *Nanomaterials*.

The author did not agree to this retraction.
